# Survival following vertebral compression fractures in population over 65 years old

**DOI:** 10.1007/s40520-023-02445-4

**Published:** 2023-05-26

**Authors:** Raquel Gutiérrez-González, Ana Royuela, Alvaro Zamarron

**Affiliations:** 1 Department of Neurosurgery, Puerta de Hierro University Hospital, IDIPHISA, Manuel de Falla 1, 28222 Majadahonda, Madrid Spain; 2 Department of Surgery, Faculty of Medicine, Autonomous University of Madrid, Arzobispo Morcillo 4, 28029 Madrid, Spain; 3 Biostatistics Unit Biomedical Research Institute-IDIPHISA, Puerta de Hierro University Hospital, Manuel de Falla 1, Madrid 28222 Majadahonda, Spain

**Keywords:** Fractures, Compression, Spinal fractures, Survival rate, Bone diseases, Metabolic

## Abstract

**Background:**

Lower mortality has been demonstrated when vertebral compression fractures (VCFs) are treated surgically (vertebral augmentation) vs. conservatively.

**Aims:**

To analyze the overall survival in patients over 65 who suffer a VCF, to review the principal causes of death, and to detect which factors are associated with a greater risk of mortality.

**Methods:**

Patients over 65 years old diagnosed with acute, non-pathologic thoracic or lumbar VCF, treated consecutively from January 2017 to December 2020, were retrospectively selected. Those patients with follow-ups under 2 years or who required arthrodesis were excluded. Overall survival was estimated by the Kaplan–Meier method. Differences in survival were tested through the log-rank test. Multivariable Cox regression was used to assess the association of covariates and time to death.

**Results:**

A total of 492 cases were included. Overall mortality was 36.2%. Survival rate at 1-, 12-, 24-, 48-, and 60-month follow-up was 97.4%, 86.6%, 78.0%, 64.4%, and 59.4%, respectively. Infection was the leading cause of death. The independent factors associated with a higher mortality risk were age, male, oncologic history, non-traumatic mechanism, and comorbidity during hospitalization. No statistical difference was found when comparing the two survival curves by treatment (vertebral augmentation vs. conservative) over time.

**Conclusion:**

Overall mortality rate was 36.2% after a median follow-up of 50.5 months (95% CI 48.2; 54.2). Age, male sex, history of oncological disease, non-traumatic mechanism of the fracture, and any comorbidity during hospitalization were identified as variables independently associated with a higher risk of mortality following a VCF in the elderly.

## Background

The longevity of the population has undergone a significant change in recent decades. In Spain, people over 65 have experienced an increase in life expectancy by about 25% in the last 30 years. Thus, the probability of surviving to increasingly advanced ages has risen, as well as the number of remaining years of life beyond those ages (63% of women aged 85 would survive in the triennium 2013–15 with 7.3 years of remaining life) [1].

Age is a well-known risk factor for fragile fractures [2]. It is estimated that the annual number of osteoporotic fractures will increase by 25% in the European Union between 2019 and 2034 [3]. The incidence of proximal femoral fractures is low between the ages of 40 and 70 but significantly increases after 75 years of age. When considering vertebral body compression fractures (VCFs), the incidence increases with age over 50, especially among women [4]. Epidemiological studies have found that 30–40% of women in their 70s were radiologically diagnosed with a VCF [5]. The ratio of symptomatic VCFs has been estimated as one-third of all vertebral fractures [6].

An excess in standardized mortality has been demonstrated in those patients that suffer an osteoporotic clinical fracture [7]. Mortality risk is highest right after the fracture but may last elevated beyond 5 years [8]. The number of fracture-related deaths in the European Union is, at least, comparable to some of the most common causes of death (lung cancer, diabetes, chronic respiratory disease) [3].

Considering VCFs exclusively, the mortality risk seems higher in symptomatic patients [8]. The hazard ratio (without adjustment for comorbidities) has been reported to be 2.8 for symptomatic patients and 1.32 for incident fractures [9, 10]. Besides that, the younger the patient is, the higher the excess mortality when the VCF occurs [11]. However, the cause of this mortality excess remains unclear. It is hypothesized that symptomatic patients are frail, with a worse general condition, and more likely to fall (which increases the risk of subsequent fractures) [9]. The impact that pain can have on the patient's quality of life may also be a determining factor. Thus, vertebral augmentation procedures have been associated with better pain control in VCFs. Reduction in excess mortality has also been evidenced when comparing these techniques with non-operative management, a protective effect that lasts up to 4 years and beyond, according to some authors [12–16]. Other hypotheses point to the beneficial impact of vertebral augmentation over pulmonary function [15].

Age, male sex, and severity of comorbidities have been associated with mortality risk after enduring an osteoporotic fracture [8, 9, 17, 18]. Other important modifiable factors are low bone mineral density (BMD) and accelerated bone loss [19]. Exercise programs have shown beneficial effects on the spinal range of motion and health-related quality of life [20, 21], though stronger evidence is required [22]. Despite being hardly documented, the recent COVID-19 pandemic impact must not be underestimated since it significantly changed population’s lifestyle. Reducing mobility and physical activity in older people might increase the risk of frailty, fractures, and mortality [23].

Studies achieved in our environment analyze epidemiology, risk factors, or hospitalization rates following VCFs [18, 24]. In fact, in Spain, osteoporosis-related vertebral fractures represent a substantial hospital burden [18]. However, long-term mortality has hardly been studied in our country. It is essential to analyze the context of the actual situation to design prevention strategies to improve patients’ care and outcomes and reduce the economic impact.

This study aims to analyze the overall survival in patients over 65 years old who suffer a VCF, to review the principal causes of death in this context, and to detect which factors are associated with a greater risk of mortality in this context and our environment.

## Methods

A single-center, retrospective study was designed to assess the survival rate in patients who suffered a VCF. The study was accomplished in a tertiary hospital with an estimated population of half a million. Our center attends about 150 patients with VCFs and 50 patients with high-energy unstable thoracolumbar fractures annually. The study was approved by the local Ethics Committee of Puerta de Hierro University Hospital and was conducted under the 1964 Helsinki Declaration and its later amendments or comparable ethical standards.

### Patients’ selection

All consecutive patients over 65 years old diagnosed with acute thoracic or lumbar VCF at levels T5–L5, without underlying oncological process, treated conservatively or surgically (vertebral augmentation procedures only), and attended at our department from January 1, 2017, to December 31, 2020, were retrospectively selected for analysis. The inclusion period was chosen according to the availability of the Department’s recordings and considering a minimum follow-up of 2 years to assess survival. Patients with incomplete follow-up (under 2 years) or who required vertebral arthrodesis due to poor progress of the fracture were excluded. Only the first process was considered in those patients who endured a second VCF during the first 3 months.

The first author (RGG) identified all patients meeting inclusion criteria according to the Department’s datasets. The first and last authors (RGG, AZ) collected the data from electronic health records and settled it in an Excel spreadsheet, anonymizing personal data.

### Fracture management

All patients diagnosed with a VCF always undergo a computed tomography to assess the integrity of the posterior wall. A magnetic resonance image is frequently acquired too. Half of the neurosurgeons in the Department treat VCFs with a brace and analgesics. In contrast, half of the doctors offer the patient the possibility to treat the fracture with a brace or vertebral augmentation procedure. Then the patient decides. According to the patients' features, the clinician recommends direct vertebral augmentation only in a few specific cases.

### Dependent variable

The date of death of patients included in the study was confirmed by the information available from the public Health System. Survival was calculated as the time from VCF diagnosis to death or end of follow-up (January 2023). The cause of death was checked through the electronic health record.

### Independent variables

Epidemiological, clinical, diagnostic, and therapeutic variables were registered and included in the dataset to ascertain any association with mortality risk. These variables included: age, gender, history of cancer, chronic steroid use, previous vertebral fracture, prior diagnosis of osteoporosis, comprehensive geriatric outpatient care, mechanism of the fracture, spinal segment affected, presence of multiple fractures at diagnosis, VCF treatment (vertebral augmentation vs. conservative management), and development of any comorbidity during hospitalization.

### Statistical analysis

Dataset information was processed and analyzed using StataCorp. 2019 (*Stata Statistical Software:*

*Release 16*. College Station, TX: StataCorp LLC). Numerical variables were represented by the mean and standard deviation (SD) or by the median and percentiles 25 and 75. Absolute and relative frequencies were used in categorical variables and as the description measure.

Overall survival was estimated by the Kaplan–Meier method, whereas differences in survival were tested through the log-rank test. Median follow-up was estimated through the Kaplan–Meier reverse method and is shown along with the corresponding 95% confidence interval (CI). Data maturity analysis was performed according to Gebski et al. [25].

Multivariable Cox regression was used to assess the association of covariates and time to death. The independent variables included were those considered relevant according to the scientific literature or the experience of the research team. Every statistical hypothesis was two-tail tested. The null hypotheses with type I error or *α* error less than 0.05 were rejected in all hypothesis contrasts.

## Results

A total of 509 consecutive patients were initially included. The incomplete follow-up excluded 14 patients who moved to another city. One patient required vertebral arthrodesis due to poor fracture evolution, and two fractures were excluded since they appeared early during the first 3 months after another event. Then 492 cases were finally analyzed.

Female prevalence was shown in the sample (72.0%), and the mean age at diagnosis was 78.9 years old (SD 7.51). Considering medical history, 23.4% of patients referred previous oncological disease, and 13.6% were under chronic steroid therapy. More than 40% of patients had a prior diagnosis of osteoporosis (40.8%), and 31.3% had suffered a vertebral fracture previously. Only 8.8% of all patients were under comprehensive outpatient geriatric care. Fractures occurred due to a traumatic mechanism (low or high energy) in 61.6% of cases, and 24.4% of the patients presented with multiple acute fractures at diagnosis. Half-fractures (49.8%) were located at the thoracic spine segment.

Most patients were treated with a brace and analgesics. Only 13.8% underwent a vertebral augmentation procedure. The median hospital stay for diagnosis and treatment was 4 days (IQR 2; 10), and 26.0% of patients developed some comorbidity during hospitalization. The most frequent pathologies, in that case, are shown in Table 1. Nine patients showed oncological comorbidity (five were first diagnosed with a malignant tumor during hospitalization, two showed progressions of known cancer, and the remaining two presented with oncological therapy-related complications).

**Table 1:** Summary of causes of comorbidity during hospitalization due to the VCF and causes of death

Causes	Co-morbidity *N* (%)	Mortality *N* (%)
Cardiac disease	22 (14.8)	14 (6.8)
Respiratory disease	13 (8.7)	13 (6.3)
Neoplasm	9 (6.0)	39 (18.9)
Infection	20 (13.4)	46 (22.3)
Neurological disease	7 (4.7)	8 (3.9)
Transplant-related complication	4 (2.7)	0 (0.0)
Other fracture	19 (12.8)	8 (3.9)
COVID-19	–	14 (6.8)
Other cause	55 (36.9)	22 (10.7)
Unknown	–	42 (20.4)

The cohort achieved data maturity at 60 months of 71%. The minimum number of subjects remaining at risk after which Kaplan–Meier survival plots for time-to-event outcomes should be curtailed was 17. Once the number remaining at risk drops below this minimum, the survival estimates are no longer meaningful in the context of the investigation. In our case, the cohort achieves 17 subjects at risk at 71 months.

**Fig. 1:**
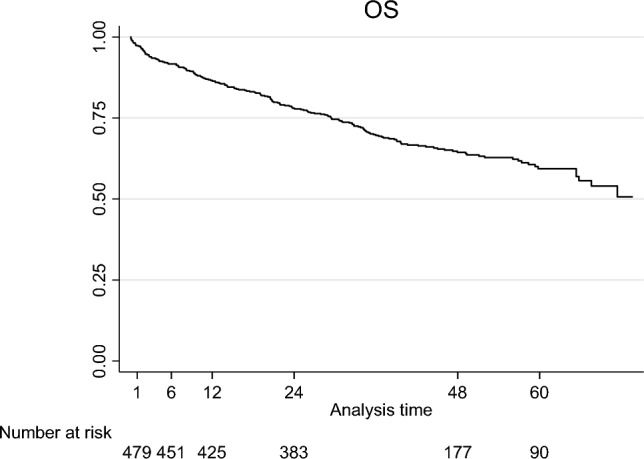
Kaplan–Meier overall survival (OS) curve for patients over 65 years old and with diagnosis of acute VCF

The median follow-up was 50.5 months (95% CI 48.2; 54.2). The overall mortality rate was 36.2%. The most frequent causes of death are shown in Table 1. Overall survival function at 1, 3, 6, 12, 24, 48, and 60 months after suffering a VCF was 97.4% months (95% CI 0.95; 0.98), 93.9% months (95% CI 0.91; 0.96), 91.7% months (95% CI 0.89; 0.94), 86.6% months (95% CI 0.83; 0.89), 78.0% (95% CI 0.74; 0.81), 64.4% months (95% CI 0.60; 0.69), and 59.4% months (95% CI 0.54; 0.64), respectively (Fig. 1; Table 2).

**Table 2:** Kaplan–Meier survivor function

Time (months)	At risk	Fail	Lost	Survivor function (%)	SE	95% CI
1	479	17	0	97.4	0.0072	0.95–0.98
3	462	11	0	93.9	0.0108	0.91–0.96
6	451	25	1	91.7	0.0125	0.89–0.94
12	424	42	0	86.6	0.0154	0.83 – 0.89
24	382	55	151	78.0	0.0187	0.74–0.81
48	171	10	77	64.4	0.0230	0.60–0.69
60	79	5	85	59.4	0.0263	0.54–0.64

*SE* standard error, *CI* confidence interval

A multivariable Cox proportional hazard model was employed to determine the association between different variables and survival. A full model was initially used. Then a stepwise backward procedure retained the age, male gender, history of oncological disease, non-traumatic mechanism of the fracture, and the presence of any comorbidity during hospitalization as variables independently associated with a higher risk of mortality following a VCF (Table 3) based on a *p* value < 0.05. All the patients that presented oncological comorbidity during hospitalization died by the end of the study.

**Table 3:** Multivariable Cox regression analysis of factors contributing to overall mortality of patients presenting an acute VCF

Covariates	Full model		Final model	
	*p* value	HR (95% CI)	*p* value	HR (95% CI)
Age	** < 0.001**	1.13 (1.10–1.17)	** < 0.001**	1.13 (1.10–1.16)
Gender male	**0.001**	1.84 (1.29–2.64)	** < 0.001**	1.89 (1.33–2.68)
History of cancer	** < 0.001**	2.40 (1.63–3.54)	** < 0.001**	2.19 (1.51–3.18)
Chronic steroid treatment	0.123	1.47 (0.90–2.40)		
Previous fracture	0.108	1.35 (0.94–1.93)		
Osteoporosis, diagnosis	0.800	1.05 (0.72–1.54)		
Outpatient geriatric care	0.585	0.87 (0.52–1.45)		
Non-traumatic mechanism	**0.025**	1.48 (1.05–2.08)	**0.004**	1.61 (1.16–2.24)
Thoracic segment	0.398	1.16 (0.82–1.65)		
Multiple fractures	0.977	0.99 (0.67–1.48)		
Vertebral augmentation	0.141	0.68 (0.41–1.14)		
Co-morbidity hospitalization	**0.001**	1.83 (1.27–2.64)	** < 0.001**	1.98 (1.40–2.81)

Statistically significant values are in bold (*p*<0.05)

*HR* hazard ratio, *CI* confidence interval

**Fig. 2:**
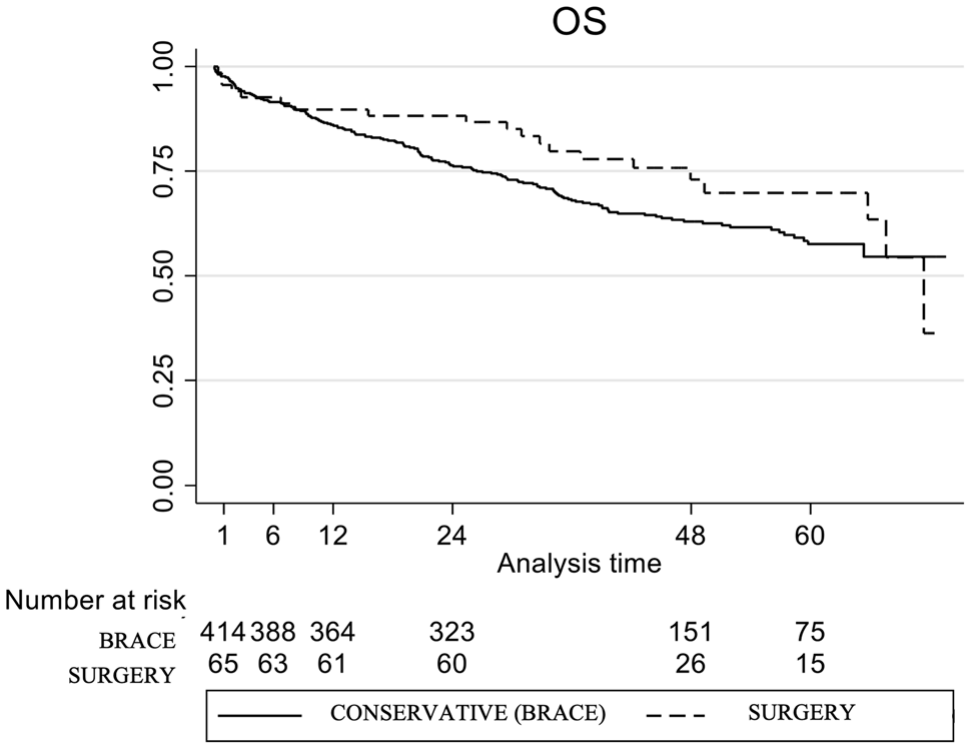
Kaplan–Meier OS curve according to the treatment received: conservative management with brace vs. surgical vertebral augmentation. Log-rank test *p* value = 0.170

No benefit was observed in those patients undergoing comprehensive outpatient geriatric care. Besides, overall survival showed no significant difference depending on fracture treatment (Fig. 2). Univariable analysis was performed to assess differences between both treatment groups, which were homogenous except for the variable “multiple fractures at diagnosis” with a lower rate in patients managed with a brace compared to surgically treated patients (22.46% vs. 36.765, respectively; *p* = 0.011).

## Discussion

The results show that one out of three patients over 65 who suffered a VCF in this sample died, considering a median follow-up of 50.5 months. Infection and cancer were the more frequent causes of death, with a meaningful 6.8% of deaths directly caused by COVID-19. Age, male sex, history of oncological disease, non-traumatic mechanism of the fracture, and any comorbidity during hospitalization were identified as variables independently associated with a higher risk of mortality following a VCF in the elderly.

There is increasing evidence of the mortality excess that a clinical osteoporotic fracture entails [8, 9]. This is the first study that analyzes VCF-related mid-term mortality in a single tertiary center in Spain. Standard fracture management includes hospitalization to complete diagnosis and treatment, which may be surgical (vertebral augmentation procedures) or conservative (analgesics and brace for at least 8 weeks). Thus, a national study showed that hospitalized osteoporotic VCFs represent 0.15% of all admissions nationwide [18]. The survival rate hereby reported shows differences from that described in other countries.

In Asia, Lee et al. [17] analyzed the Korean population aged 50 years or more, showing an overall mortality rate at 3, 6, 12, and 24 months higher in men (5.56%, 9.41%, 14.6%, and 20.61%, respectively) than in women (2.42%, 4.36%, 7.16%, and 10.48%, respectively). These data were slightly inferior to those reported in this study (overall survival of 93.9%, 91.7%, 86.6%, and 78.0%, respectively), but it must be considered that they included younger patients in their study. According to their results, the standardized mortality ratio (SMR) was highest during the first 3 months and then declined. The authors confirmed an SMR of 2.53 in men and 1.86 in women at a 2-year follow-up [17]. Another study designed by the same group reported a hazard ratio between patients with VCFs and matched controls of 1.45 in men and 1.12 in women [26].

Considering the American population, Lau et al. [9] also found that the mortality risk was higher for men than women. In their study, they included patients over 65 years, as reported in this series, with a mean age of 79.9 years (78.9 years in the present study) and female predominance in 74.4% of cases (72.0% in the study sample). Survival rates following VCF diagnosis at 3, 5, and 7 years were 53.9%, 30.9%, and 10.5%, respectively. Overall survival at 5 years was significantly higher in the present study (59.4%), but a difference in lifestyle and longevity of populations must be considered. They also evidenced that the hazard ratio between patients with VCF and matched controls was 2.17 for men and 1.72 for women [9], superior to the data reported in the Korean population [26]. Another study accomplished by the same research group posteriorly analyzed an American population aged 65 years or more and compared mortality depending on the management of the VCF [15]. Vertebral augmentation was the treatment selected in 21% of patients. Mortality risk at 4 years was superior for non-operated patients compared to those that underwent vertebroplasty and kyphoplasty procedures (49.4%, 46.2%, and 41.8%, respectively). Again, the difference with the results described in our study is relevant, with an overall mortality at 4 years of 35.62% in the present case (37.05% in non-surgical patients vs. 27.02% for those treated with a vertebral augmentation procedure). However, it is noteworthy that pathological fractures were included for analysis in this American study [15], a fact that may partially explain the differences observed.

Center et al. [27] designed a longitudinal epidemiological study in a city in Australia. They analyzed low-trauma osteoporotic fractures in subjects over 60 who asked for medical attention. Vertebral fractures associated an SMR of 2.38 in men and 1.66 in women, which is inferior to the results obtained in the Korean population [17]. They also showed that fractures in younger patients entailed decreased life expectancy compared with those in older subjects. That is, younger patients with a fracture are associated with higher mortality excess [27].

Several studies have been carried out in Europe. A study in Germany compared survival in patients over 60 years old with VCF, depending again on the therapeutic approach (16.6% were operated against 13.8% in the present study). The mean age of the sample was 79.9 years for non-operated patients and 78.2 years for those that underwent surgery. The overall survival rate at 4 years following the VCF was 65.6% for operated patients and 51.9% for those managed conservatively. In the present sample, survival at 4 years was 73.0% for surgically treaded patients and 63.0% for those who used a brace [28]. Another study performed in France with hospitalized patients over 50 years (mean age 70 years) reported an all-cause mortality rate at 12 and 24 months of 5% and 8.5%, respectively [29], compared to the 13.40% and 21.98% rates evidenced in the present study. The age of subjects must be a relevant factor, but other variables such as comorbidity, BMD, or frailty should be controlled to allow comparisons. In Spain, a randomized trial was designed to assess the effect of vertebroplasty on pain relief and quality of life. The mortality rate at the end of the study (1-year follow-up) was 8.65%, moderately inferior to that shown in the present study. In this case, the mean age of the sample was younger again (73 years) [30]. Besides that, the COVID-19 pandemic was non-existent during the study.

Thus, although the direct cause of excess mortality is unknown, frailty or poor underlying general health status may explain the increased mortality following a VCF in the elderly [18]. The most frequent causes of death in subjects over 65 years in Spain are cancer (29.9%), circulatory (28.6%) and pulmonary diseases (14.9%) in men, and circulatory disease (34%), cancer (20.1%) and mental or central nervous system pathologies (10.6%) in women [1]. However, infection was the most common cause of death in the present study (22.3% of all deaths), a relevant datum since infection is a treatable disease that may be preventable. It was followed by cancer (18.9%), cardiac (6.8%), and respiratory diseases (6.3%). COVID-19 infection accounted for another 6.8% of deaths, whereas mortality was related to another fracture in 3.9% of patients. 10.7% of deaths were due to other causes, such as gastrointestinal, renal, or metabolic diseases. In 20.4% of cases, the exact cause was unknown (patients that died out of a public hospital). These data differ from other studies published in the literature. Center et al. [27] analyzed mortality in all major types of osteoporotic fractures, and they found that death was directly related to the event in 9.5% of patients. However, most cases were hip rather than vertebral fractures. Cancer (21.9%), cardiac disease (33.3%), and stroke (18.1%) rates were comparable to those in an Australian population [27]. Choi et al. [26] focused on vertebral fractures in Korea. In their study, infection accounted for 2.4% of deaths. The most frequent causes of death were circulatory disease (25.8%), neoplasm (21.3%), and respiratory disease (10.6%), whereas trauma explained 7.9% of all cases [26]. Differences in demography and missing information in the present study prevent comparing results.

The study now reported identified age, male gender, history of oncological disease, non-traumatic mechanism of the fracture, and any comorbidity during hospitalization as variables independently associated with a higher risk of mortality following a VCF. These results are consistent with previous studies performed in different countries and populations. The mortality rate increases according to the patient’s age when the fracture occurs, as confirmed in this study [9, 11]. However, the mortality excess observed after a VCF is higher the younger the subject is compared with age-matched controls [8, 9, 11, 17, 27]. Thus, SMR in patients between 65–69 years old has been estimated at 6.41 against 1.82 in those over 85 [9]. Male sex has been associated with a higher risk of mortality too [9, 17, 18, 27], despite contradictory results in some studies [24, 31]. A sampling bias cannot be discarded since male patients hospitalized due to a VCF in our center have shown higher rates of osteoporosis than the general population in Spain (unpublished data), and low BMD has been associated with mortality risk [19]. Other authors have related this finding to a poorer health condition in men [8]. Prior history of oncological disease has also been identified with a higher risk of death since it includes recovered patients and active disease too. The risk of mortality associated with non-traumatic mechanisms may also be associated with fragile patients who debut with VCFs without trauma. The development of any morbidity during hospitalization was also identified as an independent risk factor for mortality. In the present sample, 26% of patients presented with at least one more health problem besides VCF and during hospitalization. This percentage is inferior to that reported in a nationally representative study that analyzed hospitalized osteoporotic vertebral fractures in Spain [18]. In that case, 38% of patients showed any comorbidity of potential clinical relevance, and 48% of hospitalizations were due to a disease different from the VCF. The demographic characteristics and the high socioeconomic level of the population that attended our hospital may partially influence these results. The national study abovementioned also identified factors associated with in-hospital mortality: age over 80, male gender, hospital admission not due to VCF, and the extent of comorbidity [18], which partially concur with the variables associated with increased overall mortality in the present study. All patients that presented oncological comorbidity during hospitalization in the present research died by the end of the study. This finding has not been reported before, as far as we know. However, the number of patients in this situation was small (only nine), so no relevant conclusion must be drawn.

The impact of progressive kyphosis on pulmonary function has also been hypothesized as an important factor [32]. Due to this, we analyzed if the location of the fracture in the thoracic segment was a risk factor. However, a non-significant protective effect was observed. We also evaluated the impact of comprehensive geriatric outpatient care, but no effect was confirmed. Two different viewpoints can explain this result: patients that undergo this specific standard of care are better managed but may be more fragile (or with more comorbidities), so no effect is detected; only 8.8% of the patients in the present study underwent those visits, so further studies with bigger sample size and specifically designed to assess that impact must be carried out. Other factors that may indirectly link with frailty, such as osteoporosis, chronic steroid use (a risk factor for osteoporosis), previous VCF, or multiple fractures at diagnosis, did not show any association with increased mortality. This latter result differs from a recent study that found a relationship between risk of mortality and subjects with three or more vertebral fractures [31].

Finally, the benefit of vertebral augmentation over non-surgical management regarding excess mortality in VCFs has been well-documented. Moreover, several studies have demonstrated that balloon kyphoplasty is superior to vertebroplasty [12–16]. A recent meta-analysis including more than 2 million patients showed that patients undergoing vertebral augmentation were 22% less likely to die than those with a conservative management of their VCF [16]. Pain control and early mobility may be critical factors. We analyzed this variable too, and even though augmentation procedures showed less mortality than conservative management, a significant effect was not demonstrated. Further studies with a larger size must be accomplished since only 13.8% of the patients underwent a vertebral augmentation technique in this sample.

Three main limitations must be outlined. The first refers to the sampling bias that entails considering only those patients that consulted at our hospital. It is well-known that many VCFs are not symptomatic enough to attend the hospital or may go unnoticed. The second limitation is the sample size, as we have above mentioned. Then a more significant proportion of patients undergoing vertebral augmentation procedures and comprehensive geriatric outpatient care might be recommendable for a more accurate analysis. The last limitation is related to the retrospective design of the study, a fact that restricts the quality and quantity of the data collected. Thus, an important issue to be considered is the medical condition or general health status of the patient, which should be better recorded with any tool, such as the Charlson comorbidity score, to allow comparison with other studies. Finally, the COVID-19 pandemic’s impact on diagnosis and outcome cannot be undervalued. During 2020, the number of VCFs attended at our center was inferior to those of the other years analyzed (especially during March, April, and May). Besides that, regarding the 42 patients with an unknown cause of death, 5 (11.9%) died during the national lockdown (mid-March to mid-June 2020). It is difficult to assess the real impact that the pandemic has transcended on lifestyle, frailty, and mortality, particularly in the elderly.

## Conclusion

Overall, mortality reached 36.2% in patients over 65 who suffered an acute non-pathologic VCF and were attended at our center, after a median follow-up of 50.5 months. Infection was the leading cause of death. Age, male gender, history of oncological disease, non-traumatic mechanism of the fracture, and any comorbidity during hospitalization were independently associated with a higher risk of mortality.

No benefit was observed in those patients undergoing comprehensive outpatient geriatric care. Besides, overall survival showed no significant difference depending on fracture treatment (vertebral augmentation vs. bracing) Other factors that may indirectly be associated with frailty, such as osteoporosis, chronic steroid use, previous diagnosis of vertebral fracture, or multiple fractures, did not correlate with increased mortality.

## Data Availability

The dataset generated and analyzed during the current study is available in Zenodo, https://doi.org/10.5281/zenodo.7738365, and from the corresponding author on reasonable request.
